# The level and determinants of COVID-19 vaccine acceptance in Ghana

**DOI:** 10.1371/journal.pone.0270768

**Published:** 2022-07-08

**Authors:** Grace Adjei Okai, Gordon Abekah-Nkrumah

**Affiliations:** Department of Public Administration and Health Services Management, University of Ghana Business School, Legon, Accra, Ghana; Health Directorate, LUXEMBOURG

## Abstract

**Objective:**

As part of the efforts to curb the COVID-19 pandemic, the government of Ghana has received several shipments of approved vaccines, and administration has begun in the country. Studies examining the determinants of COVID-19 vaccine acceptance in Ghana were mostly conducted before the vaccination exercise. Vaccine acceptance decisions however vary with time and hence, peoples’ decisions may have changed once vaccines became accessible. This study examines the level and determinants of COVID-19 vaccine acceptance among adult Ghanaians during the vaccination exercise.

**Methods:**

The study was a cross-sectional online survey involving Ghanaian adults (18 years and above) eligible to take the COVID-19 vaccine. The study was conducted from 18^th^ May 2021 to 14^th^ July 2021 and the questionnaire was answered by 362 respondents. Snowball sampling technique was utilized to obtain the respondents. Probit regression analysis was used to identify factors influencing COVID-19 vaccine acceptance.

**Key findings:**

Only 62.7% of the respondents indicated that they will accept the COVID-19 vaccine if provided. The regression results revealed that the decision to accept the COVID-19 vaccine was influenced by occupation, perceived susceptibility, perceived benefits and attitudes towards the vaccines.

**Conclusion:**

The findings suggest that government must implement strategies to enhance positive attitudes toward vaccines, increase the risk perception of contracting the virus and also educate the populace about the benefits of the vaccine.

## Introduction

The coronavirus disease (COVID-19) which first emerged in China in December 2019 has now affected almost every country across the world [1]. The COVID-19 pandemic has resulted in a huge burden of mortality and economic hardship worldwide [2,3]. The challenges imposed by the pandemic are more pronounced in developing countries due to the restricted number of health care facilities available, high incidence of poverty as well as lack of access to vaccines [4–6]. The unequal distribution of vaccines results in increased hospital admissions, deaths and a high risk for the emergence of new variants in developing countries [7,8].

To control the spread of the virus, several countries across the world instituted measures such as the mandatory wearing of facemasks in public places, lockdowns, social distancing, closure of schools, airports, borders and hospitality and entertainment industries [9]. Although these measures helped to reduce the spread of the virus, they had negative physical and psychosocial effects on people as well as economic hardships in several countries [10–12].

The development and deployment of a vaccine have been suggested as one of the most promising strategies to deal with the pandemic [13]. Empirical evidence shows that vaccines have been a successful measure in disease prevention for decades [14–18]. For example, it is estimated that annually, three million deaths are prevented globally due to vaccinations [19]. In countries such as the United States of America, Canada, Germany and Chile, the introduction of Haemophilus Influenza type b (Hib) vaccines has helped to eliminate Hib in these countries [20]. Additionally, vaccines have successfully helped in the eradication of smallpox which mainly affected low and lower-middle-income countries [21]. Vaccines have indeed been instrumental in improving health outcomes and life expectancy by controlling and preventing infectious diseases [22,23].

Several pharmaceutical companies have worked assiduously to develop safe and effective vaccines to keep the outbreak of the COVID-19 pandemic under control. One major obstacle facing the deployment of these vaccines is acceptability among the population, which may hinder the ability to achieve acquired immunity in a sufficient proportion of the population [24].

A study conducted by Lazarus et al. [25] to assess the potential level of acceptance of the COVID-19 vaccine across 19 countries showed that only 71.5 percent of the respondents indicated that they would accept vaccines if proven safe and effective. However, the responses varied across countries. It is therefore necessary to understand and address the wide variation in the decision to accept COVID-19 vaccines. This is due to the fact that differences in vaccine coverage between countries could delay the control of the pandemic globally and also slow down economic recovery [25].

Like developed countries, there have been numerous studies published on COVID-19 vaccine acceptance in Sub-Saharan Africa with varying acceptance rates [25–33]. High COVID-19 vaccine acceptance rates among the general public have been found in several countries such as Ecuador (97%), Malaysia (94.3%), Indonesia (93.3%), China (91.3%), Brazil (84%), Canada (80%), India(74.5%), South Korea (79.8%), Mexico (76.3%), Saudi Arabia (64.7%), Turkey (66%), Israel (75%), United Kingdom (64%), Zambia (92%), South Africa (82%) and United States of America (67%) [13,25,27,34–40,41]. These high rates of potential acceptance of COVID-19 vaccines may create the tendency to believe that vaccine acceptance is not an issue of concern. However, the picture in Africa is quite different and also, evidence suggests that the economic context of a country influences the decision of people to accept COVID-19 vaccines [42,43]. Very low acceptance rates have been reported in some studies. For example, a study conducted in the Democratic Republic of Congo among healthcare workers reported a vaccine acceptance rate of 29% [44], whereas in Cameroon, a 15.4% vaccine acceptance rate was reported among the general population [45]. Similarly, in North- Central Nigeria, Reuben et al. [46] reported a 29% acceptance rate of COVID-19 vaccines. This highlights the need to conduct more studies in Africa to address the issue of COVID-19 vaccine hesitancy.

Data from the Ghana Health Service COVID-19 dashboard indicate that as of 14^th^ January 2022, there were 152,729 confirmed cases of COVID-19 with 1,336 deaths. To mitigate the spread of the virus in Ghana, the country first received vaccines (AstraZeneca) from the United Nations-backed COVAX initiative and the administration of the same started with some priority groups. The vaccination was to be implemented in four phases with the first phase targeting health personnel, security personnel, persons with underlying conditions and adults 60years and above; Phase 2 targeted students, teachers, executive and legislature, civil servants, other essential service providers and journalists; Phase 3 involved other residents of the country who were above 18 years while Phase 4 targeted pregnant women and children less than 18 years. Phase 4 was intended to be implemented only after confirming the safety of the vaccine in the specified group [47].

After the first consignment, Ghana has received several shipments of COVID-19 vaccines (AstraZeneca, Sputnik V, Pfizer-BioNtech, Moderna, Johnson & Johnson) with administration ongoing across the country [48–50]. However, not much is known about the acceptance of the COVID-19 vaccines among the general population. Existing studies in Ghana have examined the intention of the general population [32,33,51,52] and health workers [53,54] to vaccinate against COVID-19. However, these studies were conducted during the period vaccines had not been rolled out in Ghana. There is evidence to suggest that vaccine acceptance decisions change over time [25], and hence it is likely that the attitudes of people towards COVID-19 vaccines and their decision to accept the vaccines may have changed once the vaccines became available and the level of anxiety reduced. The current study fills the gap in the literature by examining the level and determinants of COVID-19 vaccines during the vaccination rollout.

### Theoretical foundation

The decision to adopt a health-related behavior can be explained by several theoretical models which include but are not limited to the Theory of Planned Behavior [55], Health Belief Model [56], Anderson’s behavioral model [57] and Protection Motivation Theory [58]. The Health Belief Model is the most widely used in understanding vaccination-related behavior [59–61]. However, in this study, the Theory of Planned Behavior [55] was combined with the Health Belief Model [56]. This is because the Health Belief Model cannot sufficiently predict vaccination behaviour [62]. The two models and how they are used in this study are explained below.

### Health Belief Model

The Health Belief Model (HBM) posits that decision-making about health behaviors is motivated by perceived susceptibility of illness, perceived severity of illness, perceived benefits of engaging in preventive behavior, perceived costs or barriers to the behavior and cues to action [63]. The model argues that individuals will take action to prevent illness if they see themselves at risk of a condition (disease); if they believe it would have possible serious outcomes; if they believe that a particular course of action available to them would minimize the risk or severity or lead to other positive effects, and if they recognize few negative attributes related to the health action [64].

Additionally, specific cues such as factors in one’s environment can influence the final action one takes and these factors can be internal or external, ranging from experiencing symptoms of an illness to exposure to a campaign [65]. Also, self-efficacy which refers to an individual’s perception of his or her competence to successfully perform a behavior has been suggested as an HBM construct [64]. However, in this study, self-efficacy was not assessed because it has been found to be unnecessary in understanding simple health behaviors [66]. Even though the model has been used extensively, it has several limitations. One limitation of the model is its inability to take into account environmental and social forces that influence behavior [67]. Additionally, the HBM has low predictive power [68]. Hence, it is important the model is integrated with other models that are better at predicting behaviour [69].

### Theory of Planned Behaviour

The Theory of Planned Behavior [55] posits that a person’s behavior is a function of his/her intention to engage in the behavior which is determined by three factors namely attitudes, subjective norms and perceived behavioral control [55].

Attitude towards the behavior refers to the degree to which a person has a favorable or unfavorable evaluation of the behavior of interest [55]. Attitudes comprise of behavioral beliefs and evaluations of the outcome. Subjective norms refer to beliefs about whether friends or people of importance to the person will approve or disapprove performance of the behavior [62]. Perceived behavioral control refers to a person’s perception of the ease or difficulty in performing the behavior [70]. This study did not include perceived behavioral control given that availability of vaccines, timing, cost, and prioritization of COVID-19 vaccination would largely be determined by the government. Hence, individuals are unlikely to control COVID-19 vaccination. The theory of planned behavior has a high explanatory power than the health belief model [71,72].

**Fig 1:**
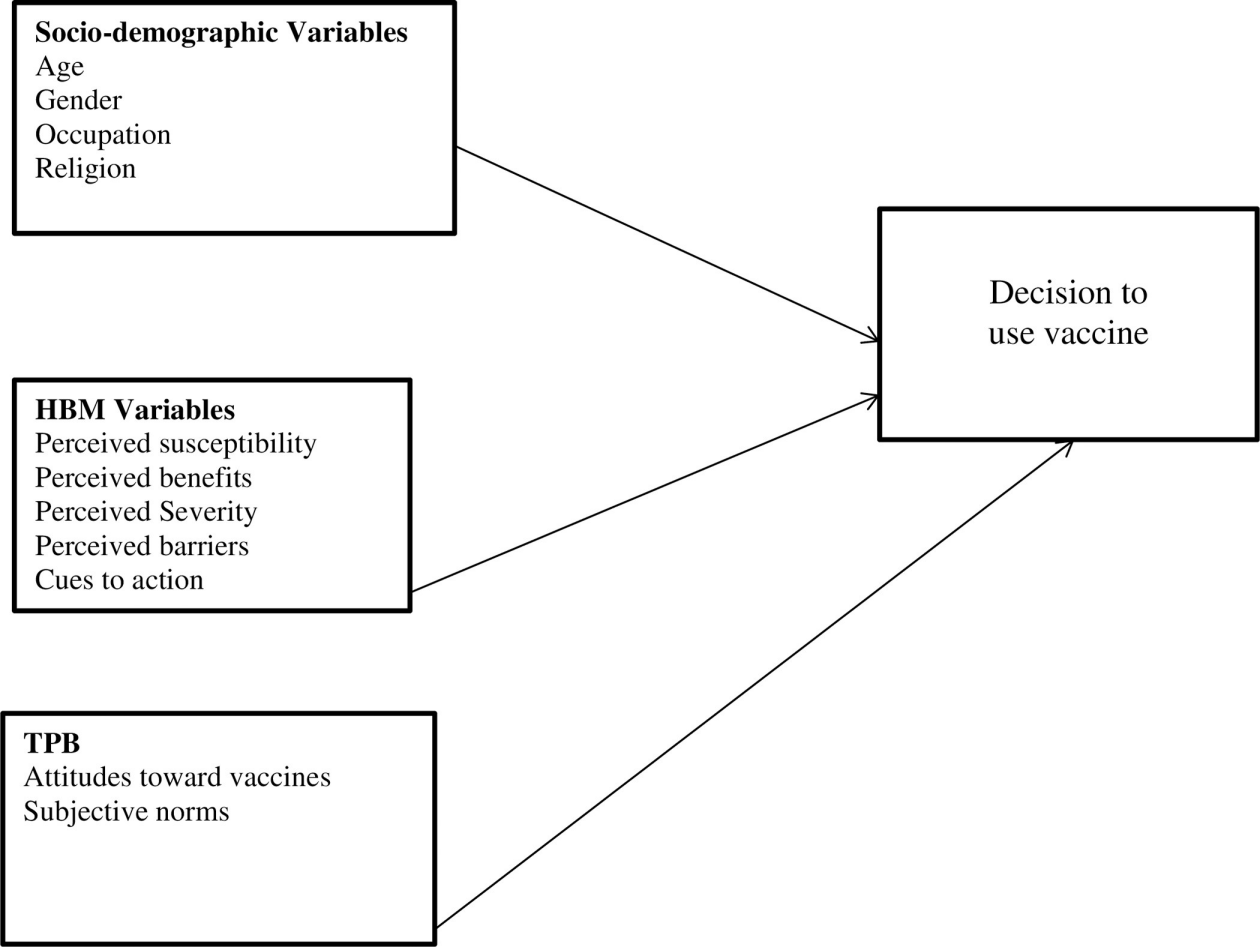
Hypothesized predictors of decision to accept COVID-19 vaccines or not.

For the purpose of the current study, the two models were combined as shown in Fig 1 to better understand those set of factors that determine the decision by individuals to either accept the COVID-19 vaccine or not.

As per Fig 1, attitudes towards vaccines is a predictor of vaccination intention. Existing studies indicate that individuals with positive attitudes towards vaccines have the intention to receive COVID-19 vaccines [73,74]. Also, persons who perceived that it is important to take the vaccine to protect others from COVID-19 and also considered the vaccines to be safe were more willing to take the vaccine [75,76]. In view of the above, we hypothesize that:

*Hypothesis 1*: *Attitudes toward vaccines significantly predict acceptance of* COVID-19 *vaccines*.

A person’s perception of social pressure to vaccinate influences the intention to engage in vaccination. When friends or parents discuss the benefits of vaccination and encourage others to accept vaccination, people are more likely to accept vaccination. Similarly, a person who thinks that his/her friends will approve of vaccination is more likely to have the intention to engage in the behavior. This has been confirmed in existing studies. For example, a study conducted by Yang [62] found that people who felt greater social pressure to accept the H1N1 vaccine were more likely to accept the vaccine. Also, Quinn et al. [77] indicated that people who had the intention to vaccinate against flu were of the view that important people around them expected them to do so. Additionally, there is evidence to suggest that subjective norms significantly predict intention to vaccinate against hepatitis A, B and human papillomavirus [78]. Deducing from the literature, we argue that:

*Hypothesis 2*. *Subjective norms significantly predict COVID-19 vaccine acceptance*.

Several studies have indicated that a person’s susceptibility to a disease influences actions such an individual will undertake. For instance, Al-Mohaithef & Padhi [34] found that persons who perceived a higher risk of being infected with the virus were more likely to accept vaccines than those with a perceived low risk. Similarly in Kuwait, individuals who perceived that they had a higher chance of contracting the virus were more willing to accept the vaccine than others [79]. However, in Hong Kong, perceived susceptibility did not significantly influence COVID-19 vaccine acceptance [80]. Hence we hypothesize that:

*Hypothesis 3*. *Perceived susceptibility significantly predicts acceptance of COVID-19 vaccines*.

Again, the perception of the severity of the disease has been found to influence the intention to accept vaccines. Empirical studies have indicated that individuals who perceive that they have a higher likelihood of experiencing complications should they contract COVID-19 have a higher intention to receive the vaccine than others [9,73,80]. Another study examining the intention of individuals to get a vaccine against zika virus found perceived severity as a significant predictor of intention to accept vaccination [81]. However, in instances where individuals perceive that the disease poses little risk, perceived severity may not influence the intention to accept vaccines. For instance, perceived severity did not significantly predict intention to accept the novel H1N1 vaccine [59]. In view of the evidence in the literature, we argue that:

*Hypothesis 4*. *Perceived severity significantly predicts acceptance of COVID-19 vaccines*.

The intention to accept vaccines is also influenced by knowledge of the effectiveness of the vaccine [82]. A study conducted by Guidry et al. [74], found that persons who perceived that COVID-19 vaccines will confer benefits were more willing to accept the vaccines than others. Similarly, Wong et al. [41] found perceived benefit as a significant predictor of intention to accept COVID-19 vaccines. Therefore, the following hypothesis is proposed:

*Hypothesis 5*. *Perceived benefits significantly predict acceptance of COVID-19 vaccines*

Evidence in the literature suggests that individuals who perceive a higher barrier to taking vaccines have a reduced likelihood of taking vaccines. Adverse effects of vaccines and negative news about vaccines make people reluctant to accept vaccines. Fall et al. [83] indicated that perceived barriers negatively influenced the intention to accept influenza vaccine. Yang [62] also found that perceived barriers significantly predicted the intention to receive a flu vaccine. Based on findings from Fall et al [83] and Yang [62] this study hypothesizes that the higher the perception of barriers, the less likely an individual will accept the COVID-19 vaccine. The hypothesis is as follows:

*Hypothesis 6*. *Perceived barriers significantly predict acceptance of COVID-19 vaccines*

Another significant predictor of intention to accept vaccines is cues to action. Recommendations from government and health experts have been shown to significantly predict intention to accept vaccines. An empirical study by Reiter et al. [84] found that healthcare provider recommendation was a significant predictor of intention to vaccinate against COVID-19. Another study by Wong et al [80] indicated that recommendations by the government significantly influenced the intention to accept COVID-19 vaccines. Based on these studies, we hypothesize that:

*Hypothesis 7*. *Cues to action significantly predict acceptance of COVID-19 vaccines*

Aside from TPB and HBM related factors, socio-demographic factors have been found to influence the intention to accept vaccines. For example, in Congo, Nzaji et al. [44] reported gender and occupation as factors influencing the potential acceptance of the COVID-19 vaccine among healthcare workers. The authors indicated that males were more likely to accept the vaccine than females, which may be due to increased risk perception of the disease in men than women. The study also indicated that doctors were more likely to accept COVID-19 vaccines than other healthcare workers. Consistent with the findings of Nzaji et al [44], lower intention to vaccinate has been identified among females in China [85], France [86] and Europe [87].

Age has also been found to be a significant predictor of intention to accept COVID-19 vaccines. Shmueli [73] and Wang et al. [88] found in their studies that about 90 percent of adults aged 65 years and above were more willing to accept the COVID-19 vaccine compared to those younger. We therefore propose that:

*Hypothesis 8*. *Socio-demographic characteristics significantly predict COVID-19 vaccine acceptance*.

## Methods

### Study population

The study population comprised of all Ghanaian adults (males and females) aged 18 years and above living in the country at the time the study was conducted. Pregnant women, children below 18 years and adult foreign residents were excluded from the study. The initial part of the questionnaire was used to screen for the eligibility of the respondents.

### Sampling and sample size

Snowball sampling method was employed in this study. Google forms was used to design an online self-administered questionnaire and it was disseminated through WhatsApp to the investigators’ contacts. Further, participants were encouraged to forward the online survey link to their contacts. This method was adopted because it ensures appropriate social distancing and eliminates movements of researchers which are very necessary to curtail the spread of the COVID-19 pandemic.

Cochran’s formula [89] was used to calculate the sample size for the study. The formula states that:

$$n_{o}=\frac{z^{2}pq}{e^{2}}$$

Where e is the desired precision level, p is the proportion of the population (potential COVID-19 vaccine acceptance rate), q is 1-p and Z is the z-value found in the Z table. In this study, a potential vaccine acceptance rate of 54% from a previous study [33], a z-value of 1.96 at 95% confidence level; and a margin of error of 0.05 were used to reach a sample size of 382. Finally, a non-response rate of 10% was assumed to have a sample size of 420.

### Data collection

A structured questionnaire developed from previous literature sources [9,41,73,75,90,91] was used for the study. The questions were worded in English and the questionnaire was pretested to ensure the internal validity of the test items. The questions were sectioned into sociodemographic characteristics, knowledge and experience of COVID-19, acceptability of COVID-19 vaccines and factors influencing the decision to accept COVID-19 vaccines. Information on sociodemographic characteristics such as gender, age, religion, education, marital status and occupation were solicited from respondents. The next section asked participants whether they had heard about COVID-19, where they first heard it and whether they had experienced it before. Questions on the acceptability of COVID-19 vaccines focused on whether or not respondents would accept the vaccines and the reasons for refusal. Regarding factors influencing the decision to accept vaccines, HBM variables (such as perceived susceptibility, perceived benefits, perceived severity, perceived barriers and cues to action) and TPB variables (such as attitudes and social norms) were measured using questions on a five-point Likert scale. Items in each of the HBM and TPB variables had a Cronbach Alpha of more than 0.6 which indicates good reliability.

The survey was conducted between 18th May 2021 and 14^th^ July 2021. In all, a total of 362 persons completed the online survey.

### Ethical consideration

Ethical approval was granted by the Dodowa Health Research Centre Institutional Review Board, with the protocol number DHRCIRB/053/05/21. An informed consent form was introduced at the beginning of the questionnaire which respondents were supposed to read and agree to before answering the questionnaire.

### Variable definition

The dependent variable of interest is the decision to accept COVID-19 vaccines. The variable is defined as follows:

Decision to accept COVID-19 vaccines: This is coded as 1 if a respondent intends to accept the COVID-19 vaccine or 0 if otherwise.

Consistent with existing literature, several independent variables were included in the study. The definition and measurement of the independent variables are shown below:

- Gender: this is coded as 0 if the respondent is a male and 1 if female.
- Age of the respondent in years.
- Religion: this is categorized as 1 if the respondent is a Christian and 0 if he/she belongs to other religions.
- Occupation: this is coded as 0 if the respondent is a healthcare worker,1 if other professional worker (e.g. accountant, lawyer, auditor, etc), 2 if others (e.g. masons, carpenters, traders, etc) and 3 if unemployed.
- Perceived Susceptibility: questions measuring this variable are aggregated for each respondent by recoding strongly agree and agree as 1 and labeled as perceived risk (i.e those who felt that they were at risk of COVID-19) and neutral, disagree and strongly disagree as 0 and labeled as No perceived risk.
- Perceived Severity: responses to questions for this variable are aggregated by recoding strongly agree and agree as 1 and labeled as severe (i.e individuals perceived that should they contract COVID-19, it will be severe) and neutral, disagree and strongly disagree as 0 and labeled as not severe.
- Perceived Benefits: items that measured this variable are aggregated by coding strongly agree and agree as 1 and referred to as beneficial (i.e considered vaccination against COVID-19 as beneficial). Neutral, disagree and strongly disagree responses were recoded as 0 and considered not beneficial.
- Attitudes towards vaccines: Responses to items measuring this variable are aggregated by categorizing strongly agree and agree as 1 and named positive (i.e respondent has a positive attitude towards COVID-19 vaccine). Other responses such as neutral, disagree and strongly disagree are categorized as 0 and labeled negative (i.e respondent has negative attitudes towards COVID-19 vaccines).
- For perceived barriers, cues to action and subjective norms, an additive index of the scores of underlying variables used to capture them was used.

### Estimation strategy

The data collected was cleaned, checked for errors and analyzed using STATA version 14.0. Frequencies, percentages of variables and other descriptive statistics were calculated.

Given that the choice to accept a vaccine is mostly a binary choice, the probability that a person would accept the vaccine or not can be modeled in a binary choice form as in the equation below: where V_j_ = 1 if an individual accepts the vaccine and V_j_ = 0 if otherwise. X denotes a vector of independent variables, Ɛ is the error term and β a vector of coefficients to be estimated.

## Results

### Socio-economic characteristics of the respondents

The socio-economic characteristics of respondents are captured in Table 1. More than half (61.9%) of the respondents were males and majority of the respondents (59.4%) were single. Less than half (36.7%) of the respondents had a Bachelor’s degree, while 30.7% were holders of a postgraduate degree. However, 19.1% had a secondary level education, whereas 9.1% and 4.1% were holders of HND/diploma and primary school/JHS leavers respectively.

**Table 1:** Socio-economic characteristics of respondents (n = 362).

Characteristic	Frequency	Percent
**Gender**		
Male	224	61.9
Female	138	39.1
**Age Range**		
18–23	80	22.1
24–29	84	23.2
30–35	119	32.9
36–41	41	11.3
42–47	22	6.1
48+	16	4.4
(Mean, Standard deviation)	30.1(9.0)	
**Marital status**		
Married	144	39.8
Single	215	59.4
Widowed	2	0.6
Divorced	1	0.2
**Level of education completed**		
No education	1	0.3
Primary school/JHS	15	4.1
Secondary school/A’level	69	19.1
HND/diploma	33	9.1
Bachelor degree	133	36.7
Post graduate	111	30.7
**Ethnic group**		
Akan	217	59.9
Ewe	57	15.8
Ga/Dangme	39	10.8
Other	49	13.5
**Occupation**		
Health workers	112	30.9
Other professional workers	109	30.1
Unemployed	77	21.3
Others	64	17.7
**Religion**		
Christianity	340	93.9
Others	22	6.1

More than half (59.9%) of the respondents were Akan, 15.8% were Ewe and 10.8% were Ga/Dangme and the remaining constitute other ethnic groups.

The results also show that 30.9% of the respondents were health workers. About 30% of the respondents were other professional workers (e.g lawyers and accountants), 21.3% were unemployed and the remaining constituted retired individuals and those engaged in other forms of employment.

About 94% of the sampled respondents were Christian, with the remaining belonging to other religions. Also, the mean age for the sampled respondents was 30.1 years.

### Covid-19 knowledge, experience and general health

About 7% of the respondents indicated that they had contracted COVID-19 before as shown in Table 2. Majority of the respondents (95.9%) indicated that they had heard of COVID-19. Among those who had ever heard of COVID-19, majority (28.5%) indicated that they first heard of COVID-19 on television. Few respondents (3.5%) first heard of the COVID-19 pandemic from friends/family and other sources. Internet, radio and social media sources constituted 28.2%, 18.2% and 21.6% respectively.

**Table 2:** COVID-19 knowledge, experience and general health(n = 362).

Variable	Frequency	Percent
**Ever heard of COVID-19?**		
Yes	347	95.9
No	15	4.1
**If Yes, where did you first hear of COVID-19?**		
Friends/ family members	12	3.5
Internet	98	28.2
Radio	63	18.2
Social media	75	21.6
Television	99	28.5
**Have you ever contracted COVID-19?**		
Yes	25	6.9
No	337	93.1
**Overall Health Rating**		
Fair	17	4.7
Good	129	35.6
Very Poor	1	0.3
Very good	215	59.4

Again, more than half (59.4%) of the respondents rated their overall health as very good. The proportion of respondents who rated their health as good and fair accounted for 35.6% and 4.7% respectively.

### Acceptability of COVID-19 vaccines

Table 3 shows that 37.3% of the respondents specified that they will not vaccinate against COVID-19 if the government provides the vaccine for free within the next 12 months. When asked about the reasons why they will not take the vaccine, majority of the respondents (63.7%) mentioned that they were not confident in the safety of the vaccines, whereas 23.7% indicated that they want to wait for a while until it seems safe to take the vaccine. Other reasons for not taking the vaccines include the perception that the vaccines will cause sterility (3.7%), belief that COVID-19 does not exist (3%) and lack of belief in vaccination (5.9%).

**Table 3:** COVID-19 vaccine acceptability(n = 362).

Variable	Frequency	Percent
**If COVID-19 vaccine is provided for free in the next 12 months, will you receive it?**		
Yes	227	62.7
No	135	37.3
**Reasons for not taking vaccines**		
Does not believe in vaccination	8	5.9
Does not believe covid-19 exist	4	3.0
Not confident in the safety of the vaccine	86	63.7
Will wait until it seems safe to take the vaccine	32	23.7
Vaccine will make me sterile	5	3.7

### Determinants of COVID-19 vaccine acceptance

A probit model using a robust covariance matrix was used to estimate the determinants of an individual’s decision to accept a COVID-19 vaccine. The robust covariance matrix was used to ensure that estimated coefficients were efficient. The marginal effect estimates of the probit model are shown in Table 4.

**Table 4:** Probit regression of factors influencing decision to accept COVID-19 vaccines.

Variables	dy/dx	Std Error	P>|z|
Female	-0.0062	0.0408	0.879
Age	0.0005	0.0117	0.966
Age^2^	0.0001	0.0002	0.629
Religion(Christian = 1)	-0.0036	0.0798	0.652
Occupation			
Other professional workers	-0.0464	0.0496	0.350
Others	-0.1953	0.0629	0.002***
Unemployed	-0.1720	0.0735	0.019**
Susceptibility(highPerceived risk = 1)	0.1922	0.0505	0.000***
Severity (severe = 1)	0.0634	0.0468	0.175
Benefit(Beneficial = 1)	0.2436	0.0504	0.000***
Attitude(Positive Attitude = 1)	0.3190	0.0723	0.000***
Barriers	-0.0073	0.0065	0.262
Cues to Action	0.0102	0.0070	0.146
Subjective norm	-0.0038	0.0092	0.682
Number of obs = 362 Wald chi2(14) = 106.90 Prob > chi2 = 0.000 Pseudo R2 = 0.3862 Log pseudolikelihood = -146.75			

Source: *results from survey data (2021)* ^***^significant at 1% ^**^ significant at 5% *significant at 10%.

The results show that occupation, perceived susceptibility, Perceived benefits and attitudes towards the vaccines significantly determine the acceptance of COVID-19 vaccines.

## Discussion

The study employed the Theory of Planned Behaviour and Health Belief Model to examine the level and determinants of COVID-19 vaccine acceptance in Ghana. The results of the study suggest that most of the respondents (62.7%) indicated that they will accept COVID-19 vaccines if the government provides them within the next 12 months. This rate is much higher than the 32.7% reported in Northern, Southern and Central Jordan [90] and other African countries such as Cameron [45], Northern Nigeria [46] and Congo [44] which reported 15.4%, 29% and 27.7% respectively. However, higher rates have been recorded in countries such as Ecuador (97 percent), Malaysia (94.3 percent), Indonesia (93.3 percent) and China (91.3 percent) [26].

The rate recorded in this study is much lower than the 83% reported in a similar study conducted in Ghana [52] prior to the initiation of vaccine administration in the country. However, much lower acceptance rates (51% and 54%) have been recorded in other studies conducted in Ghana among the general population [32,33]. Agyekum et al. [54] also found that only 39% of health care workers in Ghana intended to receive the COVID-19 vaccines. The variation in willingness to accept the COVID-19 vaccines may be due to negative social influences and also, reported side effects of the vaccines. It is also possible that the fear that was associated with getting infected with the virus and its consequences was much higher pre-vaccine deployment than at the time data was collected for this study, when the first and second waves in Ghana had already been experienced and active cases had dropped significantly. This may be an indication that vaccine hesitancy may increase during periods when the populace has a lower infection risk perception. For example, anecdotal evidence in Ghana suggests that willingness to be vaccinated increased with the identification of the Omicron variant and surge in the number of new cases getting close to the Christmas of 2021.

For most of the respondents who indicated that they will not accept the vaccine, concerns about the safety of the vaccines were a major reason for their decision. This is consistent with the findings of [53,54,92] where healthcare workers cited concerns about the safety of vaccines as the main reason for their reluctance to accept COVID-19 vaccines. This may be due to the speed at which the vaccines were developed and produced and reported side effects, such as headaches, fatigue and muscle pain associated with COVID-19 vaccines [93]. Hence, education campaigns on vaccine safety need to be strengthened in order to convince people to accept the vaccines. Additionally, even though more than half the respondents indicated that they would accept the vaccine, it will be difficult to achieve herd immunity if widespread acceptance of vaccines is not achieved.

As per the results, socio-economic characteristics such as age and gender did not significantly influence willingness to accept the COVID-19 vaccine. This contradicts findings in other studies where it has been suggested that gender [44,79,87] and age [34,73] significantly predict acceptance of COVID-19 vaccines. Additionally, being unemployed was found to significantly reduce the probability of COVID-19 vaccine acceptance. This may result from the fact that these individuals are mostly at home and hence, they may perceive that they are unlikely to contract the COVID-19 disease. Again, the results indicated that being a professional worker other than a healthcare worker reduces the probability that a person will accept the vaccine. This may be because these individuals believe that the threat and consequences of the COVID-19 pandemic are over-exaggerated and hence the vaccine may not be as important as one is made to believe.

As expected, perceived susceptibility was found to positively increase the probability of acceptance of COVID-19 vaccines. This is consistent with findings from Saudi Arabia [34], Bangladesh [94] and Kuwait [79]. This finding is not surprising given that individuals who perceive that they are likely to contract a disease will take the necessary steps to prevent it and hence are more likely to accept the COVID-19 vaccines than those who think they are unlikely to contract it.

Perceived benefit was also found to positively influence covid-19 vaccine acceptance, which corroborates the findings of other studies [74,80,95]. This is straightforward because people are more likely to accept vaccines if they know or believe that it is going to offer some form of protection in terms of reducing the severity of the symptoms should they contract the disease or decrease their chances of contracting the disease.

Consistent with other studies [44,74], the study suggests that having a positive attitude towards vaccines increases the likelihood that an individual will accept the vaccine. This is not surprising because when people perceive vaccines to be safe, effective and useful; they are more willing to accept them.

Similar to the existing literature in Ghana [52], perceived severity did not significantly predict the decision to accept COVID-19 vaccines. This may be due to the low numbers of recorded deaths in the country compared with other countries and hence, people may have the tendency to think that even if they contract the virus they will recover.

The study also found that perceived barriers did not have a significant influence on the decision to accept COVID-19 vaccines which is consistent with findings from other studies [52,73]. This may be due to the fact that vaccines are free and hence the cost may not prevent people from taking the vaccines.

## Conclusion

The study found occupation, perceived susceptibility, perceived benefits and attitudes towards vaccines as significant predictors of the decision to accept COVID-19 vaccines. This indicates that only four (Hypothesis 1,3,5 and 8) out of the eight hypotheses tested were found to be significant. The study shows that Ghana still has challenges with the acceptability of COVID-19 vaccines and hence, there is a need to come up with strategies to address the issue. These strategies may include educating the general public on the importance of getting vaccinated against the COVID-19 disease, providing evidence on the safety profile of the vaccines as well as providing comprehensive information on the side effects of the vaccines. Additionally, there is the need to emphasize the perceived risk of contracting the virus among the general public. Public health institutions must work with existing media outlets to educate the public on the mode of transmission of the disease as well as providing enough information to citizens to clear any doubt they may have about the existence of the virus. Further, the Ghana Health Service (state agency responsible for leading the implementation of Ghana’s COVID-19 emergency response plan) must provide records of the incidence and mortality associated with the COVID-19 disease in a manner that prevents the populace from thinking that the pandemic is over. Moreover, public health interventions should focus on educating the general populace on the benefits of the COVID-19 vaccine.

## Limitations of the study

Though the study provides valuable information on COVID-19 vaccine acceptance, it is not without limitations. The study adopted a non-probability sampling technique and hence, it hampers the generalizability of the results. The sampling technique also made it difficult to determine the response rate because the exact number of people who received the link to participate in the survey could not be determined. Future studies should consider using a probability sampling technique to sample respondents from the population and also use face-to-face interviews to reduce participant response bias. Additionally, the study was conducted via Whatsapp and hence, persons who had no access to the internet or smartphones could not participate in the study. Others who had access to smartphones and the internet but were not using Whatsapp could also not access the questionnaire.

Further, the study was a cross-sectional survey and hence, causal relationships between the dependent and independent variables could not be established. Future research should, therefore, consider longitudinal studies to ascertain how willingness to vaccinate varies with time. This would enable policymakers to make better decisions to address vaccine hesitancy in the country. Finally, given that several types of vaccines are being administered in Ghana, future studies should consider examining vaccine acceptance based on the types of vaccines.

## Supporting information

S1 Data (XLSX)
